# The *Saccharomyces cerevisiae* Histone Chaperone Rtt106 Mediates the Cell Cycle Recruitment of SWI/SNF and RSC to the HIR-Dependent Histone Genes

**DOI:** 10.1371/journal.pone.0021113

**Published:** 2011-06-15

**Authors:** Monica E. Ferreira, Kacie Flaherty, Philippe Prochasson

**Affiliations:** Department of Pathology and Laboratory Medicine, University of Kansas Medical Center, Kansas City, Kansas, United States of America; St. Georges University of London, United Kingdom

## Abstract

**Background:**

In *Saccharomyces cerevisiae*, three out of the four histone gene pairs (*HTA1-HTB1*, *HHT1-HHF1*, and *HHT2-HHF2*) are regulated by the HIR co-repressor complex. The histone chaperone Rtt106 has recently been shown to be present at these histone gene loci throughout the cell cycle in a HIR- and Asf1-dependent manner and involved in their transcriptional repression. The SWI/SNF and RSC chromatin remodeling complexes are both recruited to the HIR-dependent histone genes; SWI/SNF is required for their activation in S phase, whereas RSC is implicated in their repression outside of S phase. Even though their presence at the histone genes is dependent on the HIR complex, their specific recruitment has not been well characterized. In this study we focused on characterizing the role played by the histone chaperone Rtt106 in the cell cycle-dependent recruitment of SWI/SNF and RSC complexes to the histone genes.

**Methodology/Principal Findings:**

Using GST pull-down and co-immunoprecipitation assays, we showed that Rtt106 physically interacts with both the SWI/SNF and RSC complexes *in vitro* and *in vivo*. We then investigated the function of this interaction with respect to the recruitment of these complexes to HIR-dependent histone genes. Using chromatin immunoprecipitation assays (ChIP), we found that Rtt106 is important for the recruitment of both SWI/SNF and RSC complexes to the HIR-dependent histone genes. Furthermore, using synchronized cell cultures, we showed by ChIP assays that the Rtt106-dependent SWI/SNF recruitment to these histone gene loci is cell cycle regulated and restricted to late G1 phase just before the peak of histone gene expression in S phase.

**Conclusions/Significance:**

Overall, these data strongly suggest that the interaction between the histone chaperone Rtt106 and both the SWI/SNF and RSC chromatin remodeling complexes is important for the cell cycle regulated recruitment of these two complexes to the HIR-dependent histone genes.

## Introduction

Genomic DNA in eukaryotic cells is packaged into chromatin. The basic structure of chromatin is the nucleosome, which consists of 147 base pairs of DNA wrapped around an histone octamer containing two copies of each of the four core histones: H2A, H2B, H3, and H4 [1]. In *Saccharomyces cerevisiae* (budding yeast), altered histone gene dosage has been shown to affect DNA replication, chromosome segregation, transcription, and other processes which lead to genomic instability, cell cycle perturbation and aging [2], [3], [4]. Therefore, it is crucial that the assembly of chromatin on the replicated DNA in S phase is coordinated with the appropriate level of expression of all four core histones. Transcription of the histone genes is tightly regulated during the cell cycle and occurs during S phase to produce large quantities of new histones during DNA replication [3], [4]. The *S. cerevisiae* genome contains four loci that encode pairs of the four major core histones: *HTA1-HTB1* and *HTA2-HTB2* encode histone H2A/H2B, and *HHT1-HHF1* and *HHT2-HHF2* encode histone H3/H4 [4]. In budding yeast, the pattern of histone gene expression during the cell cycle is regulated by both positive and negative regulators. In addition to conserved upstream activation elements (UAS) in their promoters, six of the eight histone genes (*HTA1-HTB1*, *HHT1-HHF1*, and *HHT2-HHF2*) contain a negative regulatory site, named the NEG or CCR region, which is in close proximity to the UAS elements [5], [6]. The cell cycle regulated UAS elements are required to activate histone transcription at the G1/S transition through the recruitment of activators such as Spt10 and the SBF transcription factor [7], while the negative element is required for repression outside of S phase and in response to hydroxyurea (HU) which causes stalling of DNA replication forks [5], [6], [8]. In the absence of the negative element, histone mRNA levels still peak in early S-phase; however, transcription occurs inappropriately in G1, G2 and M phases [6], [8].

Several *trans*-acting factors involved in the repression of the three histone gene loci through their negative (NEG) *cis*-acting DNA sequence were identified through genetic screens. These include the evolutionarily conserved histone regulatory (*HIR*) and the histone promoter control (*HPC*) genes *HIR1*, *HIR2*, *HIR3*, and *HPC2* [9], [10]. We have previously shown that these four genes encode the proteins that stably assemble to form the HIR corepressor complex [11], [12]. The HIR complex is a histone chaperone that can assemble nucleosomes independently of DNA replication [11], [12]. The histone chaperone Asf1 copurifies with the HIR complex [11] and is also required for transcriptional repression of the HIR-dependent histone genes [13]. The HIR complex stably binds to DNA and nucleosomes without any known sequence specificity. Once bound to nucleosomes, a distinct protein/DNA complex is formed that is resistant to remodeling by SWI/SNF [12]. As the HIR complex binds to DNA without any sequence specificity, it is postulated that the NEG sequence present in the promoter of *HTA1-HTB1* loci is bound by an as yet uncharacterized DNA-binding factor that is suggested to be required for the direct recruitment the HIR complex to the histone genes [14]. The actions of the HIR complex are evolutionarily conserved, as in humans, HIRA (the homologue of Hir1 and Hir2) is required for histone H3.3 deposition outside of S phase [15], [16], [17]. HIRA is found associated with multiple proteins, including Cabin1 and Ubinuclein-1 (UBN1) which are homologous to Hir3 and Hpc2, respectively [18], [19]. Together these proteins likely assemble into a complex that is homologous to the yeast HIR complex [20].

ATP-dependent chromatin remodeling complexes are well known for their role in regulating gene transcription [21]. Two distinct chromatin-remodeling complexes of the Swi2/Snf2 family - the RSC and SWI/SNF chromatin remodeling complexes - are implicated in the regulation of histone gene transcription [22], [23]. The presence of the HIR complex at the histone genes renders their activation SWI/SNF dependent in S phase. The HIR complex has been reported to interact with SWI/SNF and is necessary for the recruitment of SWI/SNF to the histone genes [22]. The RSC nucleosome remodeling complex is also recruited to the *HTA1-HTB1* promoter in a cell cycle- and HIR-dependent manner [23]. However, the timing of RSC recruitment to this promoter is concomitant with histone gene repression outside of S phase, linking RSC's activity to transcriptional repression rather than activation. Despite this, *rsc* mutations do not affect histone gene transcription during an unperturbed cell cycle [23], [24].

A recent study by Fillingham and colleagues [25], has shown that the histone chaperone Rtt106 functions with Asf1 and the HIR complex to create a repressive structure at the core histone gene promoter. Rtt106 can assemble nucleosomes *in vitro* and *in vivo* and functions in heterochromatin silencing [26], [27], in replication-dependent nucleosome assembly [28], and has been linked to transcriptional elongation [27]. Rtt106 is recruited to the histone genes in a HIR- and Asf1-dependent manner and contributes to their repression outside of S phase. Similar to deletion of *ASF1* or genes encoding the HIR complex, deletion of *RTT106* results in nucleosome depletion at the *HTA1-HTB1* promoter region [25], suggesting that the repressive chromatin structure established by Asf1/HIR/Rtt106 is the main repressive mechanism at the histone genes. Similar to Asf1 and the HIR complex, Rtt106 is also present at the HIR-dependent histone genes throughout the cell cycle [25]. As shown before, recruitment of the chromatin remodeling complexes, RSC and SWI/SNF, is dependent on the HIR complex [22], [23]. However, since deletion of *RTT106* has the same phenotype on the histone genes as the deletion of the *HIR/HPC* genes [25], we asked whether Rtt106 could participate in the recruitment of the RSC and SWI/SNF complexes to the HIR-dependent histone genes.

In this study, we report a previously uncharacterized function of the histone chaperone Rtt106 as a factor that plays an essential role in the recruitment of the RSC and SWI/SNF chromatin remodeling complexes to the HIR-dependent histone genes. We found that Rtt106 can interact with both the RSC and SWI/SNF complexes *in vitro* and *in vivo*. We have shown by chromatin immunoprecipitation that deletion of *RTT106* prevents the recruitment of both the RSC and SWI/SNF complexes to the histone genes. Furthermore, we found that the Rtt106-dependent recruitment of SWI/SNF complex is cell cycle regulated and occurs in late G1 / early S phase in agreement with its requirement for activation of the histone genes. Together, our data suggest that the interaction between Rtt106 and both SWI/SNF and RSC is critical for the cell cycle dependent recruitment of these remodeling complexes to the HIR-dependent histone genes.

## Results

### Rtt106 physically interacts with the SWI/SNF- and RSC chromatin remodeling complexes

**Figure 1:**
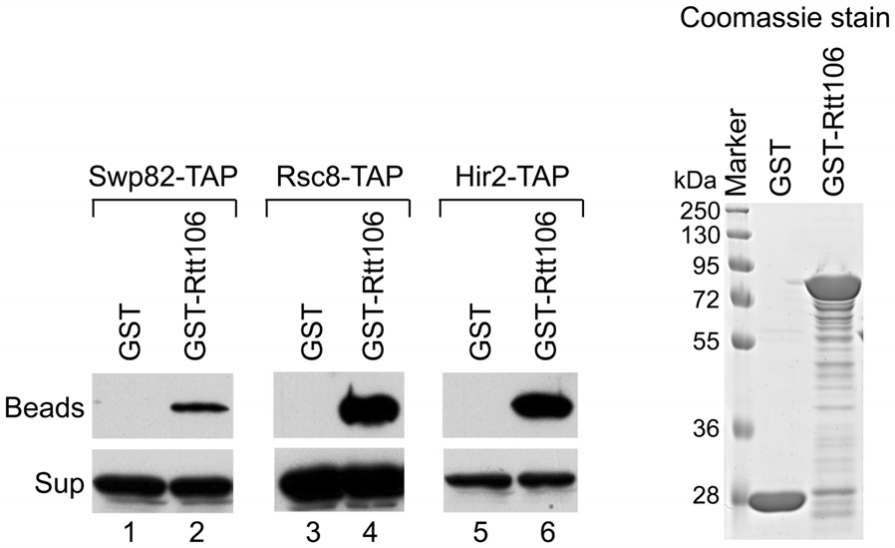
Recombinant Rtt106 interacts with SWI/SNF, RSC and HIR complexes *in vitro*. Bacterially expressed GST and GST-Rtt106 were purified and incubated with whole cell extracts from strains expressing a TAP-tagged subunit in the SWI/SNF complex (Swp82-TAP), the RSC complex (Rsc8-TAP) or the HIR complex (Hir2-TAP). Western blots to detect the TAP-tagged complexes were performed using PAP-HRP antibody.

The presence of the SWI/SNF and RSC chromatin remodeling complexes at the HIR-dependent histone genes has been previously reported to be dependent on the Hir proteins [22], [23]. Since Rtt106 is present at the histone genes throughout the cell-cycle in a HIR-dependent manner [25], we asked whether Rtt106 could participate in the recruitment of the SWI/SNF and RSC complexes to the histone genes. We first sought to determine if recombinant Rtt106 physically interacts with the SWI/SNF and RSC complexes *in vitro* by GST pull-down analysis. Purified GST-Rtt106 fusion proteins were incubated in the presence of whole-cell extracts from cells of strains containing a TAP tag sequence on a specific endogenous SWI/SNF- and RSC complex subunit gene, *SWP82* and *RSC8*, respectively (Figure 1). The presence of SWI/SNF and RSC in the supernatant and beads fractions was monitored with an antibody against the TAP tag subunits. As shown in Figure 1, both SWI/SNF (compare lanes 1 and 2) and RSC (compare lanes 3 and 4) were pulled down specifically with GST-Rtt106 in the beads fraction. This suggests that Rtt106 interacts with both SWI/SNF- and RSC-chromatin remodeling complexes *in vitro*. We also found that the TAP purified SWI/SNF complex was pulled down specifically with GST-Rtt106, suggesting that Rtt106 directly interacts with the SWI/SNF complex *in vitro* (data not shown). Furthermore, GST-Rtt106 could pull-down the Hir2-TAP subunit (Figure 1, compare lanes 5 and 6) which confirms the interaction between Rtt106 and the HIR complex which was previously reported by mass spectrometry analysis [25].

**Figure 2:**
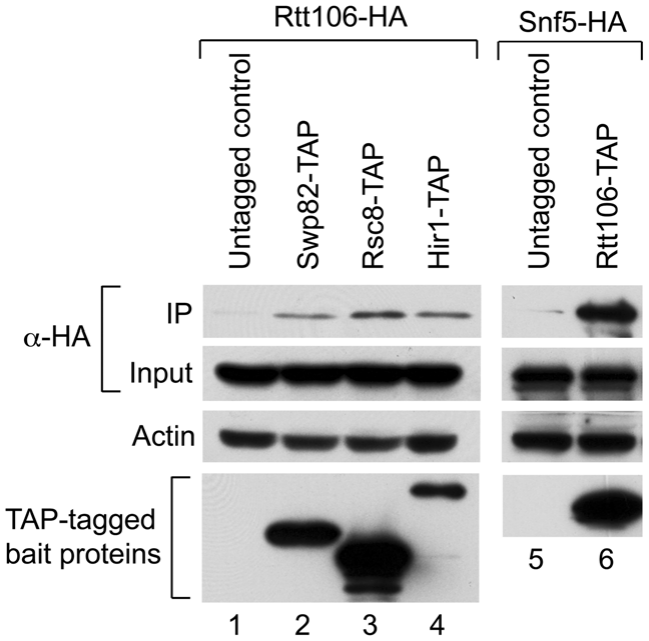
Rtt106 physically associates with RSC and SWI/SNF *in vivo*. (A) An untagged wild-type strain and strains carrying a TAP epitope tagged Swp82, -Rsc8 or -Hir1 subunit were transformed with a plasmid expressing a HA tagged Rtt106. Whole cell extracts were made and SWI/SNF, RSC and HIR complexes were immunoprecipitated in presence of ethidium bromide, using an antibody targeting the Protein A portion of the TAP tagged subunits as indicated, followed by capture on Protein A beads. Co-immunoprecipitation of Rtt106-HA was detected by western blot, using an antibody against the HA tag. Whole cell extract corresponding to 25 µg total protein per lane was used for input samples. Western blot using an antibody against endogenous actin was included as a loading control. (B) Whole cell extracts from a strain expressing TAP-tagged Rtt106 and plasmid based Snf5-HA tagged subunit, were used for immunoprecipitation of the TAP-tagged protein as described above. Co-immunoprecipitation of SWI/SNF was detected by western blot against the C-terminal HA-tag of Snf5-HA. Input and actin loading control as described in (A).

To further verify whether Rtt106 physically associates with SWI/SNF and RSC complexes in yeast, we performed co-immunoprecipitation assays with endogenous epitope tagged proteins. As shown in Figure 2, immunoprecipitated Swp82-, Rsc8-, and Hir1-TAP tagged proteins specifically associated with Rtt106-HA (Figure 2A, lanes 2, 3, and 4), while no Rtt106-HA protein was detected in an untagged strain (Figure 2A, lane 1). These results suggest that Rtt106 interacts *in vivo* with SWI/SNF, RSC and the HIR complex. We performed reciprocal immunoprecipitation, and showed that Rtt106-TAP could co-precipitate the Snf5-HA protein (Figure 2B, compare lanes 1 and 2). We also tried the reciprocal co-immunoprecipitation with Rtt106-TAP in the presence of different HA-tagged RSC subunits, but for unknown reasons, we consistently had high background with the HA-tag RSC subunits alone (data not shown); nevertheless, the GST pull down (Figure 1) as well as the co-immunoprecipitation shown in Figure 2 show a strong specific interaction between Rtt106 and RSC.

All together, these results demonstrate that Rtt106 specifically associates with the SWI/SNF and RSC chromatin remodeling complexes *in vitro* and *in vivo*. These interactions might reflect a role in the recruitment of these complexes to Rtt106 target genes such as the histone genes [25].

### The recruitment of the RSC complex to the HIR-dependent histone gene loci is dependent on Rtt106

The SWI/SNF and RSC chromatin remodeling complexes have been shown to localize to the histone genes in a HIR dependent manner and participate in their transcriptional regulation [22], [23]. Recently, Fillingham and colleagues [25] have reported that the localization of Rtt106 to *HTA1-HTB1* is dependent on Asf1 and the HIR complex and that deletion of *RTT106* results in a deregulation of the histone genes similar to *hir/hpcΔ* mutants. Since our interaction results showed that Rtt106 physically interacts *in vitro* and *in vivo* with both the SWI/SNF and RSC chromatin remodeling complexes (Figures 1 and 2), we asked whether Rtt106 could play a role in their recruitment to the histone genes. To address this question, we performed chromatin immunoprecipitation (ChIP) to monitor if the presence of SWI/SNF and RSC at the histone gene loci is dependent on Rtt106.

**Figure 3:**
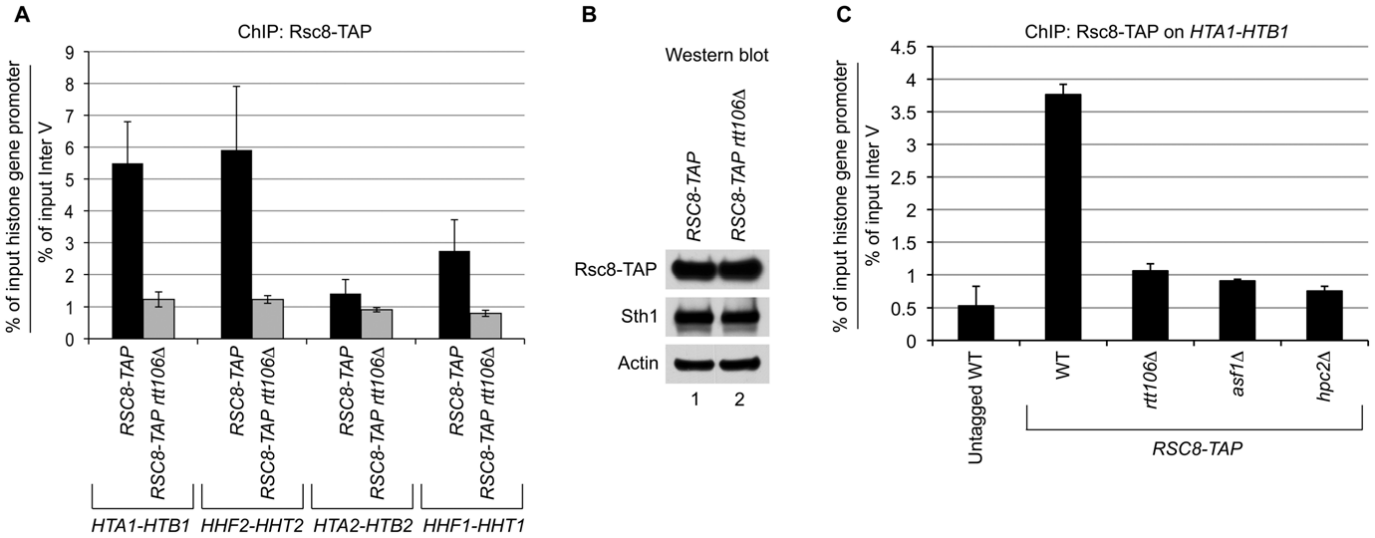
Rtt106 is essential for recruitment of RSC to the HIR-dependent histone genes. (A) Chromatin immunoprecipitation of the RSC complex were carried out as described in Material and Methods. An antibody directed against the TAP tag of the Rsc8-TAP subunit in wild-type and *rtt106Δ* strains was used. Presence of the indicated histone gene loci promoter sequence was monitored by qPCR. A non-transcribed region of chromosome V (InterV) was used as an internal background control. Error bars show the range between biological duplicates. (B) 25 µg of whole cell extract prepared from the *RSC8-TAP* (wild-type background) and *RSC8-TAP rtt106Δ* strains were loaded per lane. Protein levels of two representative RSC subunits, Rsc8-TAP and Sth1, were compared between the *RSC8-TAP* (lane 1) and *RSC8-TAP rtt106Δ* strain (lane 2) by western blots using an antibody targeting the TAP-tag in Rsc8-TAP, or an antibody against endogenous Sth1, which is the catalytic subunit of the RSC complex. Actin was used as a loading control. (C) ChIP assays were performed as in A to monitor the presence of the Rsc8-TAP tagged subunit at the *HTA1-HTB1* promoter in wild-type (WT) strain and strains deleted of *RTT106 (rtt106Δ)*, *ASF1 (asf1Δ)*, or *HPC2 (hpc2Δ)*. Enrichment of *HTA1-HTB1* promoter sequence in immunoprecipitated material was monitored by qPCR and normalized to the control region, InterV. The graph shows the average of duplicate IP reactions, and the error bars show the range between individual IPs.

We first asked if the RSC complex is recruited to the histone genes in a manner dependent on Rtt106 since we found that Rtt106 interacted with the RSC complex *in vivo* (Figure 2). We performed ChIP assays using an endogenous Rsc8-TAP-tagged subunit of the RSC complex in a wild-type and *rtt106Δ* strain and monitored its presence at all four core histone gene loci. As shown in Figure 3A, in the wild-type background (*RSC8-TAP*), Rsc8-TAP subunit localized to all three HIR-dependent histone gene loci, *HTA1-HTB1*, *HHT1-HHF1,* and *HHT2-HHF2*, as reflected by a 3- to 6-fold enrichment of these promoter sequences over the control region (Figure 3A). In contrast, deletion of *RTT106* (*RSC8-TAP rtt106Δ*) completely abrogated the recruitment of the RSC complex to these three histone gene loci as reflected by the lack of enrichment of the histone promoter sequences over the control region (Figure 3A, compare *RSC8-TAP rtt106Δ* to *RSC8-TAP*). In addition, and contrary to what has been previously shown [23], in our hands the RSC complex did not seem to localize to the *HTA2-HTB2* locus (Figure 3A); the *HTA2-HTB2* locus is not bound nor regulated by the HIR complex or Rtt106 [8], [25]. As a control, we monitored by Western blot analysis the level of two subunits of the RSC complex, Rsc8-TAP and its catalytic subunit, Sth1, in both the wild-type and *rtt106Δ* background (Figure 3B) to dismiss any indirect effects that deleting *RTT106* may have on the modulation of RSC subunits expression. We found that both subunits, Rsc8-TAP and Sth1, are expressed at the same level in wild-type and *rtt106Δ* backgrounds (Figure 3B, compare lane 1 and 2), which strongly suggests that the loss of RSC recruitment in *rtt106Δ* (Figure 3A) is a direct effect and not a consequence of a decreased protein expression level.

Additionally, as previously shown in *hir1Δ* and *hir2Δ* background [23], disruption of the HIR complex in a strain carrying a deletion of *HPC2* (*hpc2Δ*) also prevented recruitment of RSC to *HTA1-HTB1* (Figure 3C, compare *hpc2Δ* and WT). Furthermore, we found that recruitment of Rsc8-TAP subunit to the *HTA1-HTB1* promoter was also prevented in a strain deleted of *ASF1* (*asf1Δ*) (Figure 3C, compare *asf1Δ* and WT). Therefore, the lack of recruitment of the RSC complex in *hpc2*Δ and *asf1*Δ strains is consistent with the fact that Rtt106 is displaced from the histone gene promoter in their absence [25].

These results indicate that Rtt106 plays an important role in the localization of the RSC complex to the HIR-dependent histone genes. Neither the HIR complex nor Asf1 could recruit the RSC complex in absence of Rtt106 (Figure 3A and C). Furthermore, we showed that the Rtt106-dependent recruitment of the RSC complex requires both Asf1 and the HIR complex (Figure 3C). Along with the Rtt106-RSC interaction data, our results strongly suggest that Rtt106 directly recruits the RSC complex to the HIR-dependent histone genes.

### The recruitment of the SWI/SNF complex to the HIR-dependent histone genes is cell cycle regulated and dependent on Rtt106

**Figure 4:**
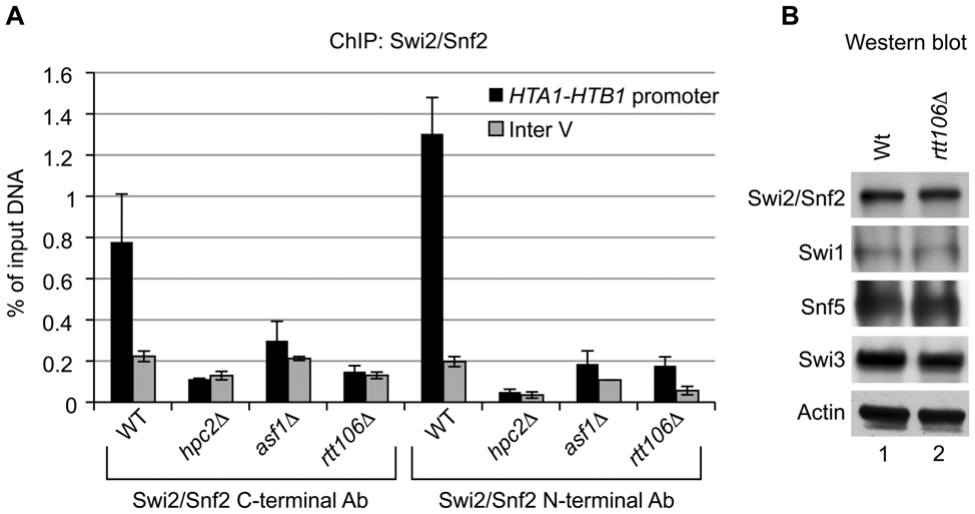
Rtt106 is required for SWI/SNF localization to *HTA1-HTB1* loci. (A) Chromatin immunoprecipitation was carried out as described in Material and Methods. Antibodies specific for the N-terminus or C-terminus of the endogenous Swi2/Snf2 subunit were used for immunoprecipitation of the SWI/SNF complex in cross-linked chromatin extracts from the wild-type strain and strains deleted of *RTT106, ASF1*, or *HPC2*. The amount of *HTA1-HTB1* promoter (black bars) precipitated was monitored by qPCR and inter V region (grey bars) was used as an internal background control from a non-transcribed region on chromosome V. Data is expressed as percentage of ChIP/Input and error bars show the range of repeated IPs. (B) 25 µg of whole cell extracts prepared from the untagged wild-type and *rtt106Δ* strains were loaded per lane. Protein levels of four SWI/SNF subunits, Swi2/Snf2, Swi1, Snf5 and Swi3, were compared between the wild-type strain (lane 1) and the *rtt106Δ* strain (lane 2) by western blot analysis using specific antibodies against each individual protein. Actin was included as a loading control.

We monitored the presence of the SWI/SNF complex at the *HTA1-HTB1* promoter by ChIP assays using two different antibodies targeting either the N-terminal or C-terminal domain of its Swi2/Snf2 catalytic subunit in wild-type, *rtt106*Δ, *hpc2*Δ, and *asf1*Δ strains (Figure 4). Strikingly, we found that deletion of *RTT106* (*rtt106Δ*) completely abolished the recruitment of SWI/SNF to the *HTA1-HTB1* promoter as shown by the loss of enrichment of the *HTA1-HTB1* promoter sequence compared to the wild-type (WT) control (Figure 4A, compare *rtt106Δ* and WT). Additionally, as previously shown [22], disruption of the HIR complex in a strain carrying a deletion of *HPC2* (*hpc2Δ*) also prevented recruitment of SWI/SNF to *HTA1-HTB1* (Figure 4A, compare *hpc2Δ* and WT). Moreover, we found that recruitment of SWI/SNF was also prevented in a strain deleted of *ASF1* (*asf1Δ*) (Figure 4A, compare *asf1Δ* and WT). Lack of recruitment of the SWI/SNF complex in *hpc2*Δ, and *asf1*Δ strains is consistent with the fact that Rtt106 is displaced from the histone gene promoter in their absence [25]. To confirm that the deletion of *RTT106* does not impair SWI/SNF recruitment indirectly by modulating the expression level of SWI/SNF subunits, we performed Western blots analysis on whole cell extracts and compared the protein level of the indicated SWI/SNF subunits in *rtt106Δ* and wild-type strains (Figure 4B). As can be clearly seen, there is no difference in protein expression for the four tested SWI/SNF subunits, Swi2/Snf2, Swi1, Snf5, and Swi3 between the wild-type and the *rtt106Δ* strain (Figure 4B, compare lane 1 and 2). This strongly suggests that the lack of SWI/SNF recruitment to *HTA1-HTB1* in *rtt106Δ* (Figure 4A) is a direct effect and not a consequence of a down regulation of SWI/SNF protein levels.

**Figure 5:**
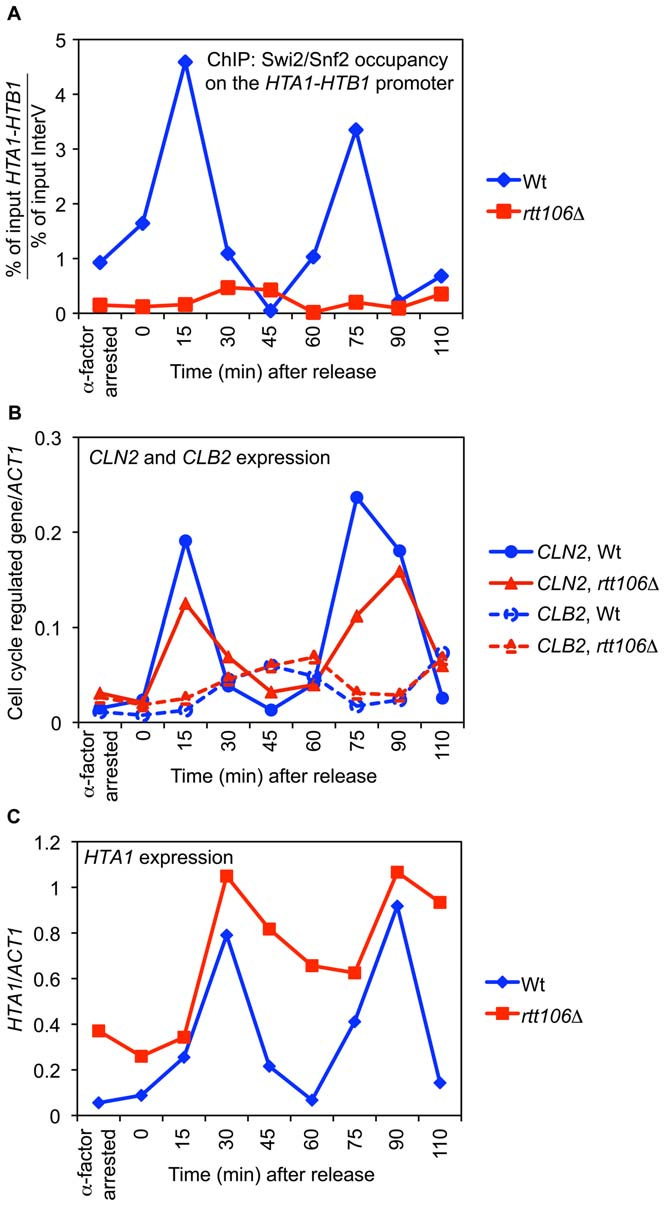
SWI/SNF recruitment to the *HTA1-HTB1* promoter is cell cycle regulated and dependent on Rtt106. (A) SWI/SNF binding to the *HTA1-HTB1* promoter in synchronized cultures of wild-type (blue line) and *rtt106Δ* (red line) strains was studied by chromatin immunoprecipitation (ChIP) assay on samples taken at the indicated time-points after release from G1 arrest induced by α-factor treatment. Samples for ChIP were cross-linked for 1h and SWI/SNF in chromatin extracts was immunoprecipitated using a specific antibody targeting the N-terminal end of Swi2/Snf2 subunit. Enrichment of *HTA1-HTB1* promoter relative to a control region (InterV) was quantified by real-time PCR. (B) Expression levels of mRNA from cell cycle regulated genes *CLN2* (solid line) and *CLB2* (dashed line) were monitored by RT-qPCR in *rtt106Δ* (red lines) and wild-type strain (blue lines) as a control for cell cycle progression and were normalized to expression of *ACT1*. Total RNA was purified from samples taken at the indicated time-points from the ChIP culture used in (A). (C) *HTA1* mRNA levels were monitored by RT-qPCR in wild-type (blue line) and *rtt106Δ* (red line) strains using the same samples used in (B) and were normalized to *ACT1* expression.

The SWI/SNF complex is required for histone gene expression in late G1 / early S phase in a HIR-dependent manner [22], where it is proposed to be necessary to overcome HIR-mediated repression of the histone genes in order to activate their transcription. Therefore, it has been predicted that recruitment of the SWI/SNF complex to the HIR-dependent histone gene loci is cell cycle regulated and happens in late G1 / early S phase just before or concomitantly with histone gene expression. However, no direct evidence has been reported to validate this theory [22]. To address this hypothesis directly, we performed ChIP assays to monitor the presence of Swi2/Snf2 at the histone genes throughout the cell cycle in α-factor synchronized cells in wild-type and *rtt106Δ* strains. As shown in Figure 5A, after release of the wild-type cells into the cell cycle from G1 arrest, we observed a strong periodic enrichment of Swi2/Snf2 occupancy at the *HTA1-HTB1* promoter in late G1 phase (Figure 5A, WT, blue line, 15 min and 75 min time points), while no enrichment was observed outside of S phase. In agreement with our data obtained in asynchronous cells (Figure 4), the absence of Rtt106 (*rtt106Δ*) completely abrogated the recruitment of SWI/SNF to the *HTA1-HTB1* loci throughout the cell cycle (Figure 5A, compare *rtt106Δ* to WT). Similar data were obtained for the other two HIR-dependent histone gene loci, *HHT1-HHF1*, and *HHT2-HHF2* (data not shown). To control for correct cell cycle progression, we monitored the expression of cell cycle regulated *CLN2* and *CLB2* genes by RT-qPCR [29] in samples taken from each time point. As shown in Figure 5B, *CLN2* expression peaked in late G1 phase (15 min and 75 min) while *CLB2* expression peaked in G2/M (45 min), no differences were observed in the cell cycle expression of these two genes between the wild-type strain and *rtt106Δ*, showing that both cell cultures were appropriately arrested and released in the cell cycle.

Finally, we monitored the level of *HTA1* expression in these samples. While *HTA1* expression fluctuated normally in the wild-type strain, peaking in S phase (30 min and 90 min) before being repressed past S phase in G2/M (45 and 60 min), deletion of *RTT106* caused constitutive expression of *HTA1* throughout the cell cycle due to the lack of proper repression outside of S phase (45 and 60 min) although peak levels were still achieved in S phase (Figure 5C). These data are consistent with previously published results [25] and similar to results obtained in strains deleted of *HIR/HPC* genes, that show how these deletions lead to constitutive expression of the histone genes, rendering them SWI/SNF independent [22].

All together, our data strongly suggest that the cell-cycle recruitment of SWI/SNF to the HIR-dependent histone gene loci in late G1 is dependent on Rtt106. In addition, our data show that SWI/SNF is recruited just before peak expression of the histone genes in S phase.

## Discussion

Our study reports novel roles for the histone chaperone Rtt106: (i) Rtt106 physically interacts with both the SWI/SNF- and RSC chromatin remodeling complexes in yeast (Figures 1 and 2); (ii) Rtt106 is essential for the localization of SWI/SNF and RSC at the HIR-dependent histone gene loci (Figures 3 and 4). Additionally, we showed that SWI/SNF is recruited to the HIR-dependent histone genes in a cell cycle dependent manner just before the peak of histone gene expression in S phase, and that this recruitment is dependent on Rtt106 (Figure 5). These findings represent important novel aspects of the biological function of the histone chaperone Rtt106 in the transcriptional regulation of the cell-cycle dependent histone genes, and strongly suggest that Rtt106 directly recruits both the SWI/SNF and RSC chromatin remodeling complexes to the histone genes in a cell cycle dependent manner.

In agreement with our data (Figure 4), the HIR complex has been previously shown to interact with SWI/SNF and is essential for the localization of SWI/SNF to the histone genes [22]. Like SWI/SNF, the RSC complex is also present at the histone genes in a HIR dependent manner [23]; however, direct interaction between RSC and the HIR complex has not been reported. More recently, Fillingham and colleagues [25] have reported that the histone chaperone Rtt106 is present at the histone genes in a HIR dependent manner. We have shown here that Rtt106 interacts with both SWI/SNF and RSC complexes (Figures 1 and 2), and that Rtt106 is essential for their recruitment to the HIR-dependent histone genes (Figures 3, 4 and 5A). Taken together, this suggests that the previously reported dependence on the HIR complex for SWI/SNF- and RSC localization to the histone genes is indirect and is actually mediated through Rtt106. In other words, deletion of *HIR/HPC* genes, which disrupts the HIR complex, leads to the displacement of Rtt106 from the histone genes, thereby preventing it from recruiting the SWI/SNF- and RSC complexes. In addition, deletion of *RTT106* does not affect the presence of the HIR complex nor Asf1 at the histone genes [25]; therefore, we can conclude from our ChIP analysis that neither the HIR complex nor Asf1 can recruit SWI/SNF or RSC at the histone genes in absence of Rtt106 (Figures 3, 4 and 5A). It is possible that the previously reported interaction between HIR and the SWI/SNF complex could be mediated through Rtt106 which was then unknown to bind either complex [22]; however, we have observed that purified HIR- and SWI/SNF complexes can interact *in vitro* (M. Ferreira and P. Prochasson, unpublished data) suggesting that *in vivo* the HIR complex could recruit SWI/SNF to other gene loci independently of Rtt106. Nevertheless, we cannot exclude that the reported physical interactions between the HIR complex, Asf1 and SWI/SNF could participate in the Rtt106-dependent recruitment of SWI/SNF complex to the histone genes by, for example, stabilizing its recruitment. Further characterization of the interaction surfaces between Rtt106/Asf1/HIR and the SWI/SNF complex is needed to make specific interacting deficient mutants and address the relative biological roles of these different interactions in recruiting, stabilizing and/or regulating the Rtt106-dependent SWI/SNF recruitment to the histone genes.

**Figure 6:**
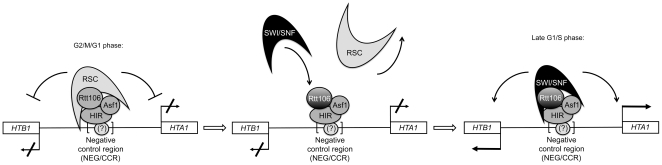
Model proposing that Rtt106 mediates the cell cycle recruitment of SWI/SNF and RSC chromatin remodeling complexes to the HIR-dependent histone genes. Based on our results demonstrating the role of Rtt106 in the recruitment of the SWI/SNF and RSC complexes, we propose the following model. Rtt106 is shown to be associated with HIR and Asf1 at the *HTA1-HTB1* promoter sequence throughout the cell cycle [25]. The indicated NEG sequence region, which is essential for HIR-mediated repression, is believed to be bound by a yet unidentified factor [14] that tethers the HIR complex and its associated factors to the promoter of the histone genes [9]. We propose that Rtt106 is the key factor of an Rtt106/HIR/Asf1 complex that mediates the cell-cycle regulated recruitment of the RSC complex outside of S phase, in G2/M and early G1 phases when histone genes are repressed and according to the previously published cell cycle regulated localization of RSC [23]. As shown by our SWI/SNF occupancy data, in late G1 phase, we propose that cell cycle specific signals trigger a switch which allows Rtt106 to recruit SWI/SNF in late G1 phase and which displace RSC from the histone gene promoter. In late G1 and S phases, SWI/SNF is shown bound to the histone gene promoter through its interaction with Rtt106 allowing transcriptional activation of the histone genes [22].

SWI/SNF is important for transcriptional activation of the HIR-dependent histone genes in S phase [22], while the presence of RSC at the *HTA1-HTB1* loci has been previously shown by ChIP analysis to be restricted to phases of the cell cycle where the histone genes are inactive (early G1, G2 and M phases) suggesting a repressive role [23]. Here we have shown for the first time that the SWI/SNF recruitment to the HIR-dependent histone gene is cell cycle regulated and restricted to late G1 phase just before peak expression of the histone genes in S phase (Figure 5). Dimova and colleagues [22] have suggested that the HIR-dependent SWI/SNF recruitment to the histone genes required for their activation could be a consequence of cell cycle regulatory signals that act on the HIR complex. A proposed model was that these signals would convert the HIR complex from co-repressor to co-activator enabling SWI/SNF recruitment and activation of the histone genes in S phase [22]. A reciprocal model could apply to explain the recruitment of the RSC complex outside of S phase [23]. However, our data, showing that Rtt106 plays an essential role in the recruitment of both SWI/SNF and RSC complexes at the histone genes, suggest that these cell cycle regulatory signals could target Rtt106 and directly modulate its interaction with the SWI/SNF and RSC complexes. Whether this is a direct effect through modification of Rtt106, or an indirect effect through modification of HIR or Asf1 remains to be explored. Identification and characterization of these cell cycle regulatory signals would further allow the understanding of the mechanism by which Rtt106 recruits the SWI/SNF and RSC complexes at specific times during the cell cycle (see model Figure 6). Another possible model is that the cell cycle regulatory signals could instead target SWI/SNF and/or RSC complexes to modulate their interactions with Rtt106 during the cell cycle. The Rtt109-mediated acetylation of histone H3 lysine 56 (H3K56ac) has also been shown to be important for activation of the histone genes in S phase ([25], [30] and P. Prochasson unpublished data), and for the localization of SWI/SNF to their promoter [30], likely by stabilizing SWI/SNF recruitment through interaction with its bromodomain. Further study will be important to gauge the relative importance of Rtt106 and H3K56ac in recruiting and stabilizing SWI/SNF to the histone genes and understand their interplay, as well as understanding the cell cycle mechanism regulating the recruitment of SWI/SNF and RSC to the histone genes. Based on previous studies [22], [23], [25] as well as our data, we propose a model in which Rtt106 constitutes the key factor of the Rtt106/HIR/Asf1 complex with respect to the cell cycle regulated recruitment of RSC outside of S phase and SWI/SNF in late G1 to the HIR-dependent histone genes (Figure 6).

Acidic transcription activators such as Gal4 and Gcn4 in yeast have been shown to interact with, and recruit, SWI/SNF to their target genes [31], [32], [33]. We have shown that two AIDs (activator interacting domain) within the Swi1- and Snf5 subunits are essential for SWI/SNF interaction and further recruitment by acidic activators to their target genes [33], [34]. Even though the C-terminal region of Rtt106 is enriched in acidic residues, we have preliminary data suggesting that Rtt106 binds to the SWI/SNF complex independently of these two AID domains (data not shown). This suggests that Rtt106 interacts with a different region or subunit(s) of the SWI/SNF complex than Gal4 and Gcn4 acidic activators and would represent a novel class of SWI/SNF recruiting protein. The SWI/SNF complex is composed of 12 known subunits [35], [36], and so far the specific functions and roles of most of them remain elusive. Identification of the SWI/SNF subunit(s), as well as the RSC subunit(s), contacted by Rtt106 would be of great interest to further understand their biological role and relevance in the Rtt106 pathway.

In conclusion, our data indicate that Rtt106 is directly involved in the cell cycle dependent recruitment of the SWI/SNF chromatin remodeling complex to the HIR-dependent histone genes in late G1 phase. Based on previous data showing the cell-cycle regulated recruitment of the RSC complex outside of late G1 and S phases [23], combined with our observation that Rtt106 is required for RSC recruitment to histone promoters in asynchronous cell cultures, we can speculate that Rtt106 is also directly involved in the cell-cycle dependent recruitment of RSC to the HIR-dependent histone genes. This represents a novel function of the histone chaperone Rtt106 and allows us to propose a promising model (Figure 6) to further characterize the way in which the SWI/SNF and RSC chromatin remodeling complexes are recruited in a cell-cycle dependent manner and how they may regulate cell cycle dependent histone gene transcription.

## Materials and Methods

### Strains, plasmids and growth conditions

*Escherichia coli* strain DH5α was used for cloning and plasmid amplification. The plasmid for bacterial expression of GST-Rtt106 was made by cloning the *RTT106* open reading frame with its native stop codon into the plasmid pGEX-5X-1. Plasmids for yeast expression of C-terminal HA-tagged -Rtt106 and -Snf5 were constructed by sequential cloning of their native promoters and protein encoding sequences into pRS416 containing three HA-tag sequences, followed by the *ADH1* terminator sequence. Yeast strains used in this study are listed in Table 1. The TAP-tagged strains and the strains bearing HA-tagged protein expressing plasmids grew indistinguishably from the untagged parental strain strongly supporting that the tagged proteins are functional. Yeast cells were grown at 30°C in YPD supplemented with adenine (40 mg/L) or, for plasmid-based expression, in SD single drop-out media.

**Table 1:** Yeast strains used for this study.

Strain	Genotype	Reference
BY4741	*MATa his3Δ1 leu2Δ0 met15Δ0 ura3Δ0*	OpenBiosystem
BY4741 RTT106-TAP	*RTT106-TAP-HIS3MX6*	OpenBiosystem
BY4741 ASF1-TAP	*ASF1-TAP-HIS3MX6*	OpenBiosystem
BY4741 HIR1-TAP	*HIR1-TAP-HIS3MX6*	OpenBiosystem
BY4741 SWP82-TAP	*SWP82-TAP-HIS3MX6*	OpenBiosystem
BY4741 RSC8-TAP	*RSC8-TAP-HIS3MX6*	OpenBiosystem
BY4741 rtt106Δ	*MATa, rtt106::kanMX6*	OpenBiosystem
MF4 (BY4741 background)	*MATa his3Δ1 leu2Δ0 ura3Δ0 RSC8-TAP-HIS3MX6 rtt106::kanMX6*	This study
BY4741 asf1Δ	*asf1::kanMX6*	OpenBiosystem
BY4741 hpc2Δ	*hpc2::kanMX6*	OpenBiosystem
MF12 (BY4741 background)	*MATa his3Δ1 leu2Δ0 met15Δ0 ura3Δ0 RSC8-TAP-HIS3MX6 asf1::kanMX6*	This study
MF17 (BY4741 background)	*MATa his3Δ1 leu2Δ0 met15Δ0 ura3Δ0 RSC8-TAP-HIS3MX6 hpc2::kanMX6*	This study

### α-factor synchronization of yeast culture

Asynchronous cultures of wild-type and *rtt106Δ* strains were grown to OD_600_ ∼0.4, washed in room temperature sterile Milli-Q water, followed by resuspension in an equal volume of fresh pre-warmed YPD. α-factor added to a final concentration of 20 µM. 2 h after the initial dose, cultures were spiked with a second dose of α-factor (20 µM) and incubation in presence of α-factor continued for a total of 4 h after which cell cycle arrest was confirmed under the microscope. Release from G1 arrest was achieved by removal of α-factor containing media, followed by a wash step in one volume fresh pre-warmed media and resuspension in two volumes of YPD supplemented with Pronase (final concentration 40 µg/mL) and CaCl_2_ (final concentration 5 mM).

### GST pull-down experiments

GST and GST-Rtt106 were expressed in *E. coli* strain, BL21(DE3)pLysS, containing the corresponding plasmids, by induction of a culture at OD 1.0 with 1 mM isopropyl-β-D-thiogalactopyranoside (IPTG) at 37°C for 2 hours. Whole cell lysate were applied on Glutathione Sepharose (GE Healthcare Life Science) according to manufacturer's instructions to purify the GST-tagged proteins. The purified GST tagged proteins were quantified by Coomassie stain using a BSA standard. Yeast extracts were prepared using bead beating in 40 mM HEPES pH 7.5, 350 mM NaCl, 0.1% Tween 20, 10% glycerol, supplemented with 1 µg/mL pepstatin, 2 µg/mL leupeptin, 0.5 mM DTT, 1 mM PMSF, and then diluted down to 150 mM NaCl. Beads containing 5 µg of GST or equivalent moles of GST-Rtt106 were incubated with 2 mg of total yeast proteins for 2 h at 4°C. Beads were washed three times 150 mM NaCl extract buffer and samples prepared for western blot analysis.

### Co-immunoprecipitation experiments and Western blot analysis

Yeast whole cell extracts were prepared as described above in 40 mM HEPES pH 7.5, 150 mM NaCl, 0.1% Tween 20, 10% glycerol, supplemented with 1 µg/mL pepstatin, 2 µg/mL leupeptin, 0.5 mM DTT, 1 mM PMSF. A total of 1 mg total protein was used per immunoprecipitation reaction, in the presence of 50 µg/mL ethidium bromide to prevent indirect interactions via DNA binding. Extracts were incubated overnight at 4°C with 2 µL of rabbit anti-Protein A antibody (Sigma-Aldrich, product #P3775) which recognize the Protein A sequence of the TAP tag. 5 µL of Protein A beads (GE Healthcare Life Science, product #17-0974-01) were then added for 1 hour to capture the immunoprecipitated material. For co-immunoprecipitation in presence of Rtt106-HA we used 50 mM Tris pH 8.0, 150 mM NaCl, 1% NP40, 10% glycerol, supplemented as described above, lysis- and wash buffers, with the antibody incubation for 2 h. Beads were washed three times and samples prepared for analysis by western blot. Protein samples were resolved on SDS-PAGE and transferred to PVDF membranes (Thermo Scientific, product #88518). Rabbit PAP (Peroxidase-Anti-Peroxidase) antibody (Sigma-Aldrich, product #P1291) 1∶1000 was used for detection of the immunoprecipitated TAP tagged subunits. HRP-linked rat HA antibody (Roche, cat #12 013 819 001) 1∶1000 was used for detection of co-immunoprecipitated HA-tagged proteins. Mouse anti-actin antibody (Abcam, product #ab8224) 1∶5000, followed by an HRP-linked secondary anti-mouse antibody (Thermo Scientific, product #31430) 1∶15000, was used for detection of endogenous actin, as a loading control, in input samples. Rabbit antibody specific for the Swi2/Snf2 N-terminal was diluted 1∶5000 in 5% milk, and rabbit antibody against Swi3 diluted 1∶1000, both followed by HRP-linked donkey anti-rabbit antibody (Jackson ImmunoResearch, product # 711-035-152) 1∶15000 in 1% milk. Rabbit antibodies against Swi1 or Snf5 were diluted 1∶1000, and followed by HRP-linked goat anti-rabbit antibody (Thermo Scientific, product #31460) 1∶20000. Sth1 was detected using rabbit antibody specific for endogenous Sth1 diluted 1∶5000, followed by HRP-linked goat anti-rabbit antibody (Thermo Scientific, product #31460) 1∶15000 in 1% milk.

### Chromatin immunoprecipitation assays

Cells were grow in YPD to OD_600_ = 0.7−1. Chromatin immunoprecipitation (ChIP) experiments were performed as previously described [37]. Shearing of the chromatin was done using a Bioruptor XL from Diagenode. ChIP of the RSC complex was performed using chromatin extracts from strains containing *RSC8-TAP* and incubated with rabbit Protein A antibody, followed by Protein A beads. The SWI/SNF complex in cross-linked chromatin was immunoprecipitated using 1 µl of rabbit Swi2/Snf2 antibodies, specific for the N-or C-terminal region of Swi2/Snf2 (gift from Dr. Joe Reese, Pennsylvania State University). Relative levels of immunoprecipitated histone promoter sequences were quantified by real-time PCR, using MyiQ (Bio-Rad) and Power SYBR PCR Master Mix (Applied Biosystems, product #4367659). An intergenic region of chromosome V was used as a non-target control. Primer sequences are available upon request.

### Quantitative RT-qPCR

Cells were collected from the culture used for the synchronized ChIP assay at the indicated time points. Cells were lysed by bead beating and total RNA was purified using E.Z.N.A. Total RNA Kit I (Omega Bio-Tek, product #R6834-02). 400 ng of total RNA per reaction was used for cDNA synthesis, using High Capacity cDNA Reverse Transcription kit (Applied Biosystems, product #4368814). cDNA from the genes of interest were quantified by real-time PCR, using MyiQ (BioRad) and Power SYBR PCR Master Mix (Applied Biosystems, product # 4367659) with specific primer pairs to the indicated genes and normalized to the expression level of *ACT1*. Primer sequences are available upon request.
